# Discriminative potential of exhaled breath condensate biomarkers with respect to chronic obstructive pulmonary disease

**DOI:** 10.1186/s12995-024-00409-6

**Published:** 2024-04-04

**Authors:** Romain Freund, Jean‑Jacques Sauvain, Guillaume Suarez, Pascal Wild, Thomas Charreau, Amélie Debatisse, Kirushanthi Sakthithasan, Valérie Jouannique, Jacques A. Pralong, Irina Guseva Canu

**Affiliations:** 1 Department of Occupational and Environmental Health (DSTE), Centre for Primary Care and Public Health (Unisanté), University of Lausanne, Lausanne, Switzerland; 2 Autonomous Paris Transport Authority (RATP), Paris, France; 3 Faculty of Medicine, University of Geneva, Geneva, Switzerland; 4 SwissMedPro Health Services, Geneva, Switzerland; 5 Hôpital de La Tour, Geneva, Switzerland

**Keywords:** COPD, Respiratory, Exhaled breath condensate, Biomarker, Oxidative stress

## Abstract

**Background:**

Chronic obstructive pulmonary disease (COPD) affecting 334 million people in the world remains a major cause of morbidity and mortality. Proper diagnosis of COPD is still a challenge and largely solely based on spirometric criteria. We aimed to investigate the potential of nitrosative/oxidative stress and related metabolic biomarkers in exhaled breath condensate (EBC) to discriminate COPD patients.

**Methods:**

Three hundred three participants were randomly selected from a 15,000-transit worker cohort within the Respiratory disease Occupational Biomonitoring Collaborative Project (ROBoCoP). COPD was defined using the Global Initiative for Chronic Obstructive Lung Disease (GOLD) criteria as post-bronchodilator ratio of Forced Expiratory Volume in 1st second to Forced Vital Capacity < 0.7 in spirometry validated by an experienced pulmonologist. Discriminative power of biomarker profiles in EBC was analyzed using linear discriminant analyses.

**Results:**

Amongst 300 participants with validated spirometry, 50.3% were female, 52.3 years old in average, 36.0% were current smokers, 12.7% ex-smokers with mean tobacco exposure of 15.4 pack-years. Twenty-one participants (7.0%) were diagnosed as COPD, including 19 new diagnoses, 12 of which with a mild COPD stage (GOLD 1). Amongst 8 biomarkers measured in EBC, combination of 2 biomarkers, Lactate and Malondialdehyde (MDA) significantly discriminated COPD subjects from non-COPD, with a 71%-accuracy, area under the receiver curve of 0.78 (*p*-value < 0.001), and a negative predictive value of 96%.

**Conclusions:**

These findings support the potential of biomarkers in EBC, in particular lactate and MDA, to discriminate COPD patients even at a mild or moderate stage. These EBC biomarkers present a non-invasive and drugless technique, which can improve COPD diagnosis in the future.

**Supplementary Information:**

The online version contains supplementary material available at 10.1186/s12995-024-00409-6.

## Introduction

Chronic obstructive pulmonary disease (COPD) affects 334 million people in the world, with a global prevalence of 11.7% [1]. In Europe, the prevalence of COPD varies between 13.5% and 13.9% and is twice as high in men as in women [1]. Worldwide, COPD is the third leading cause of death, responsible for approximately 6% of total deaths [2], the second cause of disability-adjusted life-years lost [3], and the most common cause of respiratory failure. In terms of cost, the annual burden of COPD is estimated to be €38.7 billion in Europe with up to 73% of the costs related to inability to work and accounting for nearly $50 billion in US government spending in 2010 [1, 4].

Smoking is the main risk factor in the development of COPD. Besides, occupational exposures to dusts, vapors, gases, and fumes, exposure to indoor and outdoor air pollution, maternal smoking during pregnancy or early childhood, genetic and dietary factors were recently acknowledged as COPD risk factors [5–9]. In fact, in a recent report, the Global Initiative for Chronic Obstructive Lung Disease (GOLD) highlighted air pollution as a major health threat to patients living with COPD requiring actions to reduce the morbidity and mortality related to poor air quality around the world [7]. Occupational exposure is responsible for 20% of COPD overall [10], and 31% of COPD in non-smokers [11]. One third of occupationally exposed COPD patients must stop working definitively due to their respiratory problems [12].

Studies have shown that the earlier COPD patients receive treatment, the greater the recovery of pulmonary function, highlighting the importance of an early diagnosis [13]. However, 70% of COPD patients are diagnosed at advanced stages and 50% die approximately 3.6 years after the first hospitalization [14]. The diagnosis of COPD is still challenging because some patients are asymptomatic or only have mild and non-specific respiratory symptoms. Besides clinical symptoms including chronic cough (~ 3 months a year) and sputum production, spirometry is the most widely used method to diagnose COPD. Although spirometry is necessary to assess the prevalence and severity of COPD, when spirometry becomes abnormal and a decrease in FEV1 is evident, the disease has already been present for some years and has already progressed. Moreover, pre-bronchidilatation spirometry is often incorrectly used in routine medical examinations, rising concerns of diagnostic misclassification [1, 15]. In its last report, GOLD proposes a definition of “early COPD” related to the initial biological mechanisms that eventually lead to COPD which should be differentiated from a clinical “early”, which reflects the initial perception of symptoms, functional limitation and/or structural abnormalities noted [9]. Therefore, considering this screening limits of spirometry, having an easy to measure, early COPD diagnostic biomarkers at disposal could be more cost-effective from a health perspective. It could be particularly useful for screening populations at risk and targeting preventive interventions (i.e. smoking cessation, reduction of occupational exposure to pollutants…).

The oxidative stress plays a central role in the pathophysiology of COPD and could be measured using biomarkers [16–18]. Oxidative stress induces epigenetic changes resulting from direct activation of oxidative stress response genes and inflammation resulting from indirect intracellular signaling pathways, through the overproduction of reactive oxygen species (ROS) and altered gene expressions. ROS release inflammatory mediators, impairing phagocytosis of apoptotic cells and weakening the ability of corticosteroids to repress proinflammatory genes expression. Biomarkers of inflammation, lipid peroxidation, protein oxidation and DNA damage can result from tissue damage, protein alteration, and remodeling of extracellular matrix and mucus. As a consequence of long-term inflammatory stimulation, COPD may also be accompanied by modified metabolism [7, 19, 20]. This phenomenon is currently investigated from biomarkers discovery perspective with some promising results indicating usefulness of oxidative stress biomarkers to monitor COPD progression and severity [21, 22].

For that purpose, exhaled breath condensate (EBC), a noninvasively collected biological matrix, allows measuring different biomarkers representative of inflammatory processes in the lungs [23–26]. Sophisticated metabolomic approaches applied to EBC may improve the diagnosis of COPD. Indeed, COPD patients present modified concentrations of acetate, propionate and lactate [27] or formate [28]. Statistical models using these anions among others could differentiate between healthy and COPD patients. Moreover, the distribution of some of these biomarkers in EBC samples was shown to be modified in workers exposed to soapstone and quartz [29] and public transit workers [30].

In the present study, we aimed to investigate whether these biomarkers in EBC can discriminate COPD cases among a sample of underground subway workers.

## Material and methods

### Study design, setting and participants

The study sample was constructed by applying a stratified random sampling procedure to a cohort of 15,000 underground subway workers in the Parisian transport company, within the Respiratory disease Occupational Biomonitoring Collaborative Project (ROBoCoP).

The strata were defined by sex, age, smoking status (smokers, ex-smokers, non-smokers) and exposure (depending on the occupation: station agents, security guards and locomotive operators). Company occupational physician contacted one by one the randomly selected workers in each stratum and included those who accepted participation. During the inclusion call, the physician verified the inclusion criteria (i.e., being employed at the company for at least one year and older than 40 years). In case of refusal, retirement, or employment in another company, the physician called the next worker listed in the same strata, and so on, until recruiting at least 300 participants, to insure a 90%-statistical power in the analysis as determined by the sample size calculation in the study protocol [31].

All data collection procedures and recent results of associations between long-term occupational exposure to subway PM10 and prevalence of respiratory diseases conducted within ROBoCoP were published elsewhere [31–33]. Briefly, we used three main sources of individual and health data: 1-the epidemiological questionnaire completed by study participants; 2-the biomedical tests and corresponding forms completed by the research team in the field; 3-individual occupational medical records. The latter were used for cross-checking information from epidemiological questionnaire.

Participants’ characteristics collected were age, gender, height, weight, and ethnicity. Smoking status was defined as non-smoker, current smoker (at least one cigarette, e-cigarette, or shisha per day), and ex-smoker (no tobacco consumption for at least one year). Lifetime smoking exposure was calculated by multiplying the estimation of cigarette packs smoked per day by the number of years smoking. Self-declared symptoms associated with COPD collected were cough, phlegm, shortness of breath and wheezing. Medical history associated with COPD collected were asthma, bronchitis, COPD, emphysema, eczema, and pollen allergies.

### COPD diagnosis

Spirometry was performed by the trained nurses using an electronic spirometer (Easy on-PC®, ndd Medical Technologies Inc., Andover, MA) according to the American Thoracic Society/European Respiratory Society guidelines [34]. All spirometry-based results were validated by a board-certified pneumologist. All tests suggesting an obstructive syndrome were repeated after administrating a bronchodilator. The Global Initiative for Chronic Obstructive Lung Disease (GOLD) defines spirometrically confirmed COPD based on a FEV1 to a forced vital capacity (FVC) ratio smaller than 0.7 [9]. Participants having a FEV1/FVC ratio > 0.7 were classified as non-COPD. Reference values of lung function parameters, FEV1, FVC, FEF 25–75, or FEV1/FVC ratio were computed using the Global Lung Function Initiative (GLI 2012) [35]. GOLD further classifies COPD severity as Pre-COPD: FEV1/FVC ≥ 0.7 and FEV1 ≥ 80% of predicted value and respiratory symptoms (cough, phlegm); Stage 1 (mild): FEV1/FVC < 0.7 and FEV1 ≥ 80% of predicted value; Stage 2 (moderate): FEV1/FVC < 0.7 and FEV1 < 80% but ≥ 50% of predicted value; Stage 3 (severe): FEV1/FVC < 0.7 and FEV1 < 50% but ≥ 30% of predicted value; and Stage 4 (very severe): FEV1/FVC < 0.7 and FEV1 < 30% of predicted value [9]. Preserved ratio impaired spirometry (PRISm) was defined as FEV1 < 80% of predicted value and FEV1/FVC ratio ≥ 0.70 [9].

### EBC sampling, storage and analyses

EBC were collected at the RATP occupational medicine center, during 20 min of tidal breathing using Turbo-Deccs® (Medivac, Parma, Italy) and nose clips, according to the latest recommendations [25]. An average volume of 2.26 ± 0.84 ml EBC per participant could thus be collected. Participants were asked to rinse their mouth with water before the collection and swallow the saliva during sampling. All EBC samples collected in polypropylene tubes with caps were sealed immediately after collection and stored at -20 °C during collection and transportation and at -80 °C until the analysis as described in Hemmendinger M et al*.* for MDA [36] and Sauvain JJ et al*.* for anions [29].

In this paper, we selected ion chromatography as a simple and quantitative method allowing determination of different metabolites (anions) in EBC. Nevertheless, NMR-based metabolomic and e-nose techniques are promising approaches for clinical diagnostic of chronic airways diseases but not available for us at the moment [37–39].

Briefly, the seven selected anions were analysed by injecting 10 μl of the EBC sample without any treatment into a Dionex ICS 5000 + ion chromatograph, equipped with an analytical column IonPac AS11-HC250 mm, 4 μm (ThermoFisher Scientific, Ecublens, Switzerland) and a conductivity detector. The validated method gives good recoveries (comprised between 87–102%) with acceptable coefficients of variation comprised between 7–12%, except for NO_3_^−^ which reach 20% [29]. The low LOD, comprised between 0.07 and 0.58 μM (depending on the analyte), allowed the quantification of all these anions in all samples. Such detection limits are quite similar to NMR-based techniques (0.13 ± 0.03 µM based on the reference standard 3-trimethylsylil-[2,2,3,3-2H4]propionate and using the bins at 7.0–7.3 ppm) [40].

### Data management and statistical analysis

Data management and statistical analyses were performed using Stata, version 16 (College Station, Tx). Individual data from questionnaires were computerized and double-checked for correctness and completeness. The categorical variables was expressed as frequency. Continuous variables were expressed as median, interquartile ranges (IQR). Variables with missing values were sent to the participant’s occupational physician for completion. Interval regression models were used to impute censured data (below limit of detection (LOD) and comprised between LOD and limit of quantification (LOQ)) [41].

The central research hypothesis in ROBoCoP was that a combination of several biomarkers measured in EBC would be more efficient than a single biomarker for COPD diagnosis. Therefore, biomarkers with a *p*-value below 0.20 in univariate analysis were selected to be included in the multivariate analysis. To evaluate the discriminant power of different biomarkers on COPD diagnosis we used a linear discriminant analysis [42]. The standardized discriminant function coefficient of the variable indicate its discriminating ability. Relative contribution of the variable to separation of the two groups can be assessed by comparing the coefficients in the discriminant function. The canonical structure coefficients measure the correlation between the discriminating variables and the discriminant function [42]. We evaluated the classification performance in regards of error rates (leave one out table). We used receiver operating characteristic (ROC) curves and calculated area under the curve (AUC) to illustrate the discriminant power of different biomarkers on COPD diagnosis. The method of cutpoint estimation by biomarker used in the ROC curve was the Liu method maximizing the product of the sensitivity and specificity [43]. In models using multiple biomarkers, if the sum of canonical structure coefficients (β) biomarkers multiplied by their concentration was below 1, the participant was considered as no COPD, and over 1 as COPD as below.

$$Y=\sum ({\beta }_{biomarker x }\times [Biomarker x])$$

## Results

### Description of study sample

The study sample included 303 participants. Three participants were excluded for lack of spirometry results. Participants were 52-year-old in average and mostly Caucasian. The average body mass index (BMI) of participants was 25.9 kg/m^2^. Thirty-six percent were current smokers, 12.7% ex-smokers with overall mean tobacco exposure of 15.4 pack-years (Table 1).

**Table 1:** Sample description

**Variable**	**No COPD** *n* = 279	**COPD** *n* = 21	**Total** *n* = 300	***p-value***
Gender, women, n (%)	141 (50.5)	10 (47.6)	151 (50.3)	0.07
Mean age, years, m (SD)	52.2 (5.3)	53.5 (5.4)	52.3 (5.3)	0.22
Age category, n (%)				
40–45	38 (13.6)	2 (9.5)	40 (13.3)	
45–50	43 (15.4)	2 (9.5)	45 (15.0)	
50–55	91 (32.6)	7 (33.3)	98 (32.7)	
55–60	98 (35.1)	8 (38.1)	106 (35.3)	
60 +	9 (3.2)	2 (9.5)	11 (3.7)	0.55
BMI (kg/m^2^), m (SD)	26.0 (4.3)	25.3 (4.4)	25.9 (4.3)	0.67
Ethnicity, n (%)				
Caucasian	225 (80.6)	17 (81.0)	242 (80.7)	
African	44 (15.8)	4 (19.0)	48 (16.0)	
Other	4 (1.4)	0 (0.0)	4 (1.3)	0.84
Smoking category, n (%)				
Non smoker	147 (52.7)	7 (33.3)	154 (51.3)	
Current smoker	95 (34.1)	13 (61.9)	108 (36.0)	
Ex-smoker	37 (13.3)	1 (4.8)	38 (12.7)	**0.04**
Tobacco exposure, pack-years, m (SD)	14.8 (14.6)	21.5 (17.2)	15.4 (14.9)	0.11
Occupation, n (%)				0.91
Station agent	123 (44.1)	9 (42.9)	132 (44.0)	
Locomotive operator	113 (40.5)	8 (38.1)	121 (40.3)	
Security guard	43 (15.4)	4 (19.0)	47 (15.7)	
Employement duration, years, m (SD)	24.0 (6.1)	24.9 (7.5)	24.1 (6.2)	0.54
Spirometry, m (SD)				
FEV1% predicted (GLI)	99.3 (13.1)	83.6 (17.0)	98.2 (14.0)	** < 0.001**
FVC % predicted (GLI)	101.1 (12.9)	103.6 (17.8)	101.2 (13.2)	0.26
FEV1/FVC % predicted (GLI)	98.0 (5.4)	80.0 (6.8)	96.7 (7.2)	** < 0.001**
FEF 25–75% predicted (GLI)	97.7 (28.2)	48.1 (18.7)	94.2 (30.4)	** < 0.001**
COPD Stage, n (%)				
PRISm	18 (6.5)	-	-	
Pre-COPD: At-risk	63 (22.6)	-	-	
GOLD 1: Mild	-	12 (57.1)	-	
GOLD 2: Moderate	-	8 (38.1)	-	
GOLD 3: Severe	-	1 (4.8)	-	
GOLD 4: Very severe	-	0 (0.0)	-	
Self declared symptoms, n (%)				
Cough	44 (15.8)	11 (52.4)	55 (18.3)	** < 0.001**
Phlegm	41 (14.7)	6 (28.6)	47 (15.7)	0.11
Shortness of breath (Dyspnea)	40 (14.3)	4 (19.0)	44 (14.7)	0.53
Wheezing	22 (7.9)	5 (23.8)	27 (9.0)	** < 0.01**
At least one above symptom	90 (32.2)	12 (57.1)	102 (34.0)	**0.01**
Mean number symptoms above, m (SD)	0.5 (0.9)	1.2 (1.4)	0.6 (1.0)	**0.03**
At least one COPD symptom exacerbation	-	7 (33.3)	-	
Medical history, n (%)				
Asthma	27 (9.7)	3 (14.3)	30 (10.0)	0.42
Bronchitis	7 (2.5)	1 (4.8)	8 (2.7)	0.45
COPD	0 (0.0)	2 (9.5)	2 (0.7)	0.44
Emphyzema	3 (1.1)	2 (9.5)	5 (1.7)	** < 0.01**
Eczema	56 (20.1)	3 (14.3)	59 (19.7)	**0.04**
Pollen Allergies	60 (21.5)	6 (28.6)	66 (22.0)	0.78

*Abbreviations: BMI* body mass index, *COPD* Chronic obstructive pulmonary disease, *FEF 25–75%* Forced expiratory flow during the middle half of the FVC, *FEV1* forced expiratory volume in one second, *FVC* forced vital capacity, *GOLD* Global initiative for obstructive lung disease, *PRISm* preserved ratio abnormal spirometry, *SD* standard deviation

Values shown are mean (SD) or number (%) of subjects where appropriate

For definitions of GOLD stage and PRISm refer to the methods section

Associations with a p-value of < 0.05 are bolded

Twenty-one participants (7.0%) had spirometrically confirmed COPD, including 19 participants unaware of their COPD status, 12 of which with a mild COPD (GOLD 1), 8 with a moderate COPD (GOLD 2) and 1 with a severe COPD (GOLD 3). Values of last measure of participant’s spirometry, pre-bronchodilator, or post-bronchodilator in case FEV1/FVC ratio < 0.70 are presented Table 1. Thirty-four percent of the participants reported to have at least one symptom linked with COPD, *i.e.* cough, phlegm, shortness of breath and wheezing (Table 1).

COPD patients were more likely to be smokers compared to no COPD participants (current smokers 61.9% vs 34.1%) (Table 1). In spirometry, FEV1% predicted, FVC % predicted and FEF 25–75% were worse in COPD patients compared to no COPD participants. Likewise, patients with COPD declared more COPD-related symptoms compared to no COPD participants: cough (52.4% vs 15.8%, *p*-value < 0.001), wheezing (23.8% vs 7.9%, *p*-value < 0.01) with mean number of COPD-related symptoms of 1.2 versus 0.5 in no-COPD participants (*p*-value 0.03) (Table 1).

### Biomarkers measured in EBC

Eight biomarkers in EBC were analyzed for each participant: Lactate, Formate, Acetate, Butyrate, MDA, Nitrate, Nitrite and Propionate, with results available for all of them in 293 participants.

#### Malondialdehyde (MDA)

MDA concentration was higher in COPD patient compared to non-COPD with median concentration of 288.8 pg/ml [IQR 203.2–432.5] and 255.5 pg/ml [IQR 146.7–407.6], respectively (*p*-value 0.12) (Table 2). In 91.2% of samples, MDA concentration was above the LOQ (75.0 pg/ml) and 6.3% had concentration comprised between LOQ and LOD (25.0 pg/ml).

**Table 2:** Biomarker concentrations measured in EBC

**Biomarker** **Median [IQR], missing data**	**No COPD** *n* = 279	**COPD** *n* = 21	**Total** *n* = 300	***p-value***
Lactate (µmol/L)	2.4 [0.8–4.6], 6	0.4 [0.2–2.1], 0	2.2 [0.6–4.5], 6	** < 0.001**
MDA (pg/mL)	255.5 [146.7–407.6], 3	288.8 [203.2–432.5], 0	261.2 [150.4–410.6], 3	**0.12**

*Abbreviations: COPD* Chronic obstructive pulmonary disease, *IQR* Interquartile range, *MDA* Malondialdehyde

Associations with a *p*-value of < 0.05 are bolded

#### Anion patterns

Lactate concentration was lower in COPD patient compared to non-COPD with median concentration of 0.4 µmol/L [IQR 0.2–2.1] and 2.4 µmol/L [IQR 0.8–4.6], respectively (*p*-value < 0.001) (Table 2). In 84.7% of samples, Lactate concentration was above the LOQ (0.3 µmol/L) and 10.3% had concentration comprised between LOQ and LOD (0.1 µmol/L).

The concentrations of other anions were not statistically different between workers with and without COPD (Supplementary Material Table 1).

### Discriminant analysis of biomarkers

**Fig. 1:**
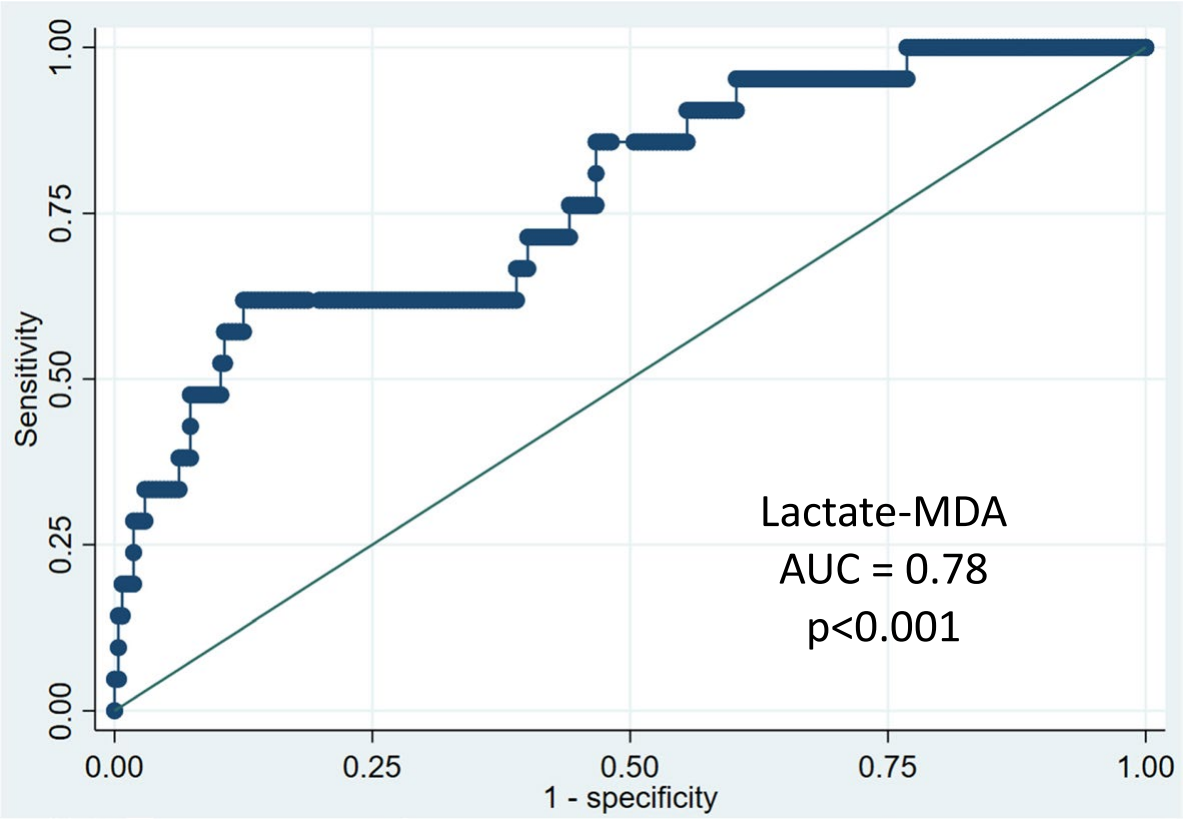
Receiver operating characteristic curve of the two-biomarker model. Notes: Linear discriminant analysis; Abbreviations: AUC Area Under the Curve, MDA Malondialdehyde

Amongst eight biomarkers measured in EBC, Lactate and MDA together discriminated COPD subjects from non-COPD (*p* < 0.001). The model with this biomarker combination had a 71%-accuracy, a 62%-sensitivity, a 71%-specificity, a 14%-positive predictive value and 96%-negative predictive value (Table 3). Using receiver operating characteristic (ROC) curve of the 2-biomarker model, the area under the curve (AUC) was 0.78 (Fig. 1).

**Table 3:** Model description and performance

	**MDA *n* = 293**	**Lactate *n* = 293**	**Lactate-MDA *n* = 293**
Linear discriminant analysis *p*-value	0.03	< 0.001	*p* < 0.001
Performance			
AUC	0.61	0.73	0.78
Sensitivity (%)	57	57	62
Specificity (%)	54	73	71
Positive Predictive value (%)	9	14	14
Negative Predictive value (%)	94	96	96
Youden Index	0.11	0.30	0.33
Prevalence (%)	7	7	7
Error rate (%)	46	28	29
Success rate (%)	54	72	71

*Abbreviations: AUC* Area Under the Curve, *MDA* Malondialdehyde

Linear discriminant analysis

The description (canonic structure) of this model is presented in Supplementary Material Table 2. The standardized discriminant function coefficients indicated that both MDA (0.610) and Lactate (-0.923) had a strong discriminating ability regarding COPD. Moreover, Lactate decrease had a higher contribution than MDA increase in our discriminant model. The canonical structure coefficients of Lactate and MDA indicated that both variables were correlated with our discriminant model (β = –0.802 and 0.427, respectively).

Individually, MDA discriminated COPD subjects from non-COPD (*p* = 0.03) with a 54%-accuracy (Table 3), an AUC of 0.61 (Supplementary Material Fig. 1) and an optimal cutoff value concentration of 239.5 pg/mL. Likewise, individually Lactate discriminated COPD subjects from non-COPD (*p*-value < 0.001) with a 72%-accuracy (Table 3), an AUC of 0.73 (Supplementary Material Fig. 1) and an optimal cutoff value concentration of 1.71 µmol/L. The complete set of biomarkers did not improve the discriminant power of the model compared to the model with two biomarkers (Supplementary Material Table 3). The description (canonic structure) of the model with 8 biomarkers is presented in Supplementary Material Table 4.

Lactate concentration and MDA concentration in EBC could not statistically discriminate pre-COPD and PRISm patients. Moreover, lactate and MDA concentrations in EBC were not correlated with FEF 25–75, the corresponding correlation coefficients were 0.0593 and -0.0806, respectively.

## Discussion

These findings support the ability of biomarkers measured in EBC to discriminate COPD patients even at a mild or moderate stage. These biomarkers represent a non-invasive and drugless technique, which can improve COPD diagnosis in the future.

Amongst eight biomarkers measured in EBC, combination of Lactate and MDA significantly discriminated COPD subjects from non-COPD. In a previous study, we showed no correlation between Lactate and MDA in EBC [30]. MDA is one of the end-products of lipid peroxidation and a well-established marker of oxidative stress [44]. These results are consistent with the literature. Indeed, COPD patients have higher MDA levels in EBC than healthy participants [45]. Likewise, in blood an increase of MDA was also reported in COPD patients compared to healthy participants and as COPD severity increases [46]. Last, an increased MDA was associated with aging and smoking [44]. In our study, COPD patients were comparable to non-COPD participants in term of age but not in term of smoking. When adjusted on smoking, our model kept its performance, highlighting its robustness and interest for COPD diagnosis.

Lactate levels in our study are comparable with the levels reported in healthy patients [29], whereas in COPD patient lactate level was significantly decreased, allowing their discrimination. COPD is a heterogeneous disease with a range of underlying mechanisms. One main feature is the thickening of airway smooth muscles coupled with an excessive production of sputum. These changes are induced by mitochondrial dysfunction with increased glycolysis [47]. Lactate results inevitably from the glycolysis and its major route of catabolism is oxidation in the mitochondria or gluconeogenesis [48]. In contrary to Michaeloudes [47], the observed decrease of Lactate in our population of COPD diagnosed patients could result from an increased use of Lactate to sustain the glycolysis process in COPD compared to the non-COPD population [48]. Gregus et al*.* also reported a decreased Lactate level in EBC of patients with different inflammatory pathologies (including COPD) compared to healthy volunteers [49]. It could also be a sign of an adaptation to the higher muscle work induced by the development of the disease. Indeed, it was reported that for trained/adapted persons, 90% of Lactate is used as energy source, whereas only 70% is used in non-trained persons [50]. Finally, Xue et al*.* demonstrated significant increase of Lactate associated with the aggravation of COPD severity and with COPD symptomatology [51]. However, in our study, we analyzed only newly diagnosed patients, mainly mild and moderate stages and paucisymptomatic.

Among the strengths, it is worth mentioning that all spirometry tests were conducted by trained nurses, validated by a board-certified pulmonologist and all tests suggesting an obstructive syndrome were repeated after administrating a bronchodilator, as recommended by GOLD [9]. In addition, we used validated methods for the analysis of these different metabolites [29, 36]. We used an interval regression models to account for censured data below limit of detection (LOD) and between LOD and limit of quantification (LOQ) as recommended [41]. Moreover, EBC sampling and spirometry were performed at the same time-point at the individual participant level, avoiding change of COPD or biomarker status in the interval. Mild and moderate stages COPD are difficult to diagnose, potentially underestimating the performance of our model in a COPD population with severe patients. Indeed, a majority of the twenty-one participants were newly diagnosed COPD, mostly mild COPD stage (GOLD 1) and paucisymptomatic. Half of them were women, known to be highly misdiagnosed with respect to COPD. Thus, in a COPD population with severe stages (GOLD 3–4), oxidative biomarker such as MDA, would increase with COPD severity [46, 52] and therefore could improve the discriminative power of our model. Interestingly, the predictive negative value of our model using Lactate and MDA was high (96%), highlighting its potential to be used as a screening tool. Besides, 36.0% of the study population were current smokers, 12.7% ex-smokers with a mean tobacco exposure of 15.4 pack-years. This shows the importance of early diagnosis and smoking cessation intervention to stop disease progression in this population. Finally, the presence of metabolites in EBC results from the combined action of multiples enzymes, reflecting the consequences of exposure to pollutants and the development of pathologies. The observed modifications in oxidative stress biomarkers concentrations combined with changes in glycolysis metabolites illustrate the interest to consider multiple biomarker approach and corresponds to a strength of this study.

The study has also limitations. First, assessing the performance of biomarkers in EBC comparing to a suboptimal reference, gives a controversial view of spirometry as a gold standard for COPD screening and diagnosis. False positive and false negative participants with EBC technique, compared to this reference test, could therefore be early COPD patients and true non-COPD participants. Follow-up of false positive participants, potentially early-COPD related to the initial biological mechanisms that eventually lead to COPD, may find rapidly new COPD diagnosis, highlighting the earliness discrimination power of our oxidative biomarkers in the course of the disease. However, the use of EBC in the course of COPD diagnosis has still to be determined. In fact, spirometry and EBC are both non-invasive methods and bronchodilation responsiveness testing is considered safe [53]. EBC needs a well-equipped laboratory, and the clinician will have results well after the collection, whereas spirometry results are immediate. Additionally, while spirometry is a standardized method, EBC collection and analysis have still many issues to be answered, as reported in the latest ATS/ERS technical standard [25]. Second, the lower COPD prevalence of 7% in our occupational study population compared to the COPD prevalence of 13.7% in the general population was expected [1]. The healthy worker effect, especially its healthy worker survivor effect (HWSE) component, might partially explain this finding. The HWSE results from a continuing selection process where workers who remain employed tend to be healthier than those who leave employment [54]. Despite the lower power of our study, we were able to find statistically significant results. Last, our study is a diagnostic accuracy cohort-type cross-sectional study [55], establishing or excluding COPD diagnosis. Even if it is a first critical step, medical decision-makers generally rely on the impact of early diagnosis on patient health outcomes, such as mortality, morbidity, and quality of life. Therefore, future longitudinal studies could more extensively address the impact of the use of these biomarkers in EBC on COPD patient health outcomes.

## Conclusion

This study supports the potential of biomarkers in EBC, in particular lactate and MDA, to discriminate COPD patients even at a mild or moderate stage. Studies have shown that the earlier COPD patients receive treatment, the greater the recovery of pulmonary function, highlighting the importance of an early diagnosis [13]. However, screening and proper diagnosis of COPD is still a challenge. We found that a combination of two biomarkers measured in EBC, Lactate a central metabolite in glycolysis, and MDA a well-established marker of oxidative stress, significantly discriminated COPD subjects from non-COPD. These biomarkers non-invasively measured in EBC, can improve COPD diagnosis in the future.

### Supplementary Information

**Additional file 1: Supplementary Material Table 1. **Eight measured biomarkers concentration. **Supplementary Material Table 2. **Description (canonic structure) of the two-biomarker model. **Supplementary Material Table 3. **Performance of the eight-biomarker model. **Supplementary Material Table 4. **Description (canonic structure) of the eight-biomarker model. **Supplementary Material Figure 1. **Receiver operating characteristic curve for each biomarker used in the model.

## Data Availability

The data are not publicly available due to ethical and privacy restrictions.
