# Supplementary material for: Discriminative potential of exhaled breath condensate biomarkers with respect to chronic obstructive pulmonary disease
Source: J Occup Med Toxicol. 2024 Apr 4;19:10. doi: 10.1186/s12995-024-00409-6 (PMC10993619; doi:10.1186/s12995-024-00409-6)
Supplement: Supplementary file 1 — Additional file 1: Supplementary Material Table 1. Eight measured biomarkers concentration. Supplementary Material Table 2. Description (canonic structure) of the two-biomarker model. Supplementary Material Table 3. Performance of the eight-biomarker model. Supplementary Material Table 4. Description (canonic structure) of the eight-biomarker model. Supplementary Material Figure 1. Receiver operating characteristic curve for each biomarker used in the model. [file 12995_2024_409_MOESM1_ESM.docx]

**Supplementary materials:**

- **Supp table 1 :** Eight measured biomarkers concentration
- **Supp table 2:** Description (canonic structure) of the two-biomarker model
- **Supp table 3:** Performance of the eight-biomarker model
- **Supp table 4:** Description (canonic structure) of the eight-biomarker model
- **Supp figure 1:** Receiver operating characteristic curve for each biomarker used in the two-biomarker model

**Supplementary materials**

**Supplementary Material Table 1:** Eight measured biomarkers concentration

| **Biomarker**  **Median [IQR], missing data** | **No COPD**  n = 279 | **COPD**  n = 21 | **Total**  n=300 | ***p-value*** |
| --- | --- | --- | --- | --- |
| Lactate (µmol/L) | 2.4 [0.8-4.6], 6 | 0.4 [0.2-2.1], 0 | 2.2 [0.6-4.5], 6 | **<0.001** |
| MDA (pg/mL) | 255.5 [146.7-407.6], 3 | 288.8 [203.2-432.5], 0 | 261.2 [150.4-410.6], 3 | **0.12** |
| Butyrate (µmol/L) | 0.9 [0.5-1.7], 6 | 0.8 [0.5-1.1], 0 | 0.9 [0.5-1.7], 6 | 0.38 |
| Nitrite (µmol/L) | 1.1 [0.7-1.8], 6 | 1.0 [0.7-1.7], 0 | 1.1 [0.7-1.8], 6 | 0.60 |
| Formate (µmol/L) | 1.5 [1.1-2.1], 6 | 1.4 [1.0-2.2], 0 | 1.4 [1.1-2.1], 6 | 0.88 |
| Propionate (µmol/L) | 9.3 [4.8-17.1], 6 | 9.8 [5.5-16.6], 0 | 9.3 [4.8-17.1], 6 | 0.98 |
| Nitrate (µmol/L) | 12.6 [8.8-15.9], 6 | 10.9 [8.4-17.7], 0 | 12.5 [8.8-16.0], 6 | 0.70 |
| Acetate (µmol/L) | 35.1 [23.2-55.5], 6 | 41.5 [21.6-55.5], 0 | 35.1 [22.9-55.5], 6 | 0.81 |

Abbreviations: COPD Chronic obstructive pulmonary disease, IQR Interquartile range, MDA Malondialdehyde. Associations with a p-value of <0.20 are bolded.

**Supplementary Material Table 2:** Description (canonic structure) of the two-biomarker model

|  | **Standardized canonical discriminant function coefficients** | **Canonical structure coefficients**  (β) |
| --- | --- | --- |
| Lactate | -0.923 | -0.802 |
| MDA | 0.610 | 0.427 |

Abbreviations: MDA Malondialdehyde

**Supplementary Material Table 3:** Performance of the eight-biomarker model

|  | **All anions**  **n=293** |  |
| --- | --- | --- |
| Linear discriminant analysis p-value | <0.001 |  |
| **Performance** |  |  |
| AUC | 0.80 |  |
| Sensitivity (%) | 67 |  |
| Specificity (%) | 75 |  |
| Positive Predictive value (%) | 17 |  |
| Negative Predictive value (%) | 97 |  |
| Youden Index | 0.41 |  |
| Prevalence (%) | 7 |  |
| Error rate (%) | 26 |  |
| Success rate (%) | 74 |  |

Notes: All anions: Lactate, Formate, Acetate, Butyrate, MDA, Nitrate, Nitrite, Propionate. Abbreviations: AUC Area Under the Curve, MDA Malondialdehyde

**Supplementary Material Table 4:** Description (canonic structure) of the eight-biomarker model

|  | **Standardized canonical discriminant function coefficients** | **Canonical structure**  **coefficients**  (β) |
| --- | --- | --- |
| MDA | 0.710 | 0.393 |
| Acetate | 0.516 | - 0.129 |
| Butyrate | - 0.254 | - 0.135 |
| Formate | - 0.087 | - 0.114 |
| Lactate | - 0.953 | - 0.739 |
| Nitrate | - 0.142 | 0.163 |
| Nitrite | - 0.429 | - 0.126 |
| Propionate | - 0.084 | - 0.089 |

Abbreviations: MDA Malondialdehyde

**Supplementary Material Figure 1 :** Receiver operating characteristic curve for each biomarker used in the model


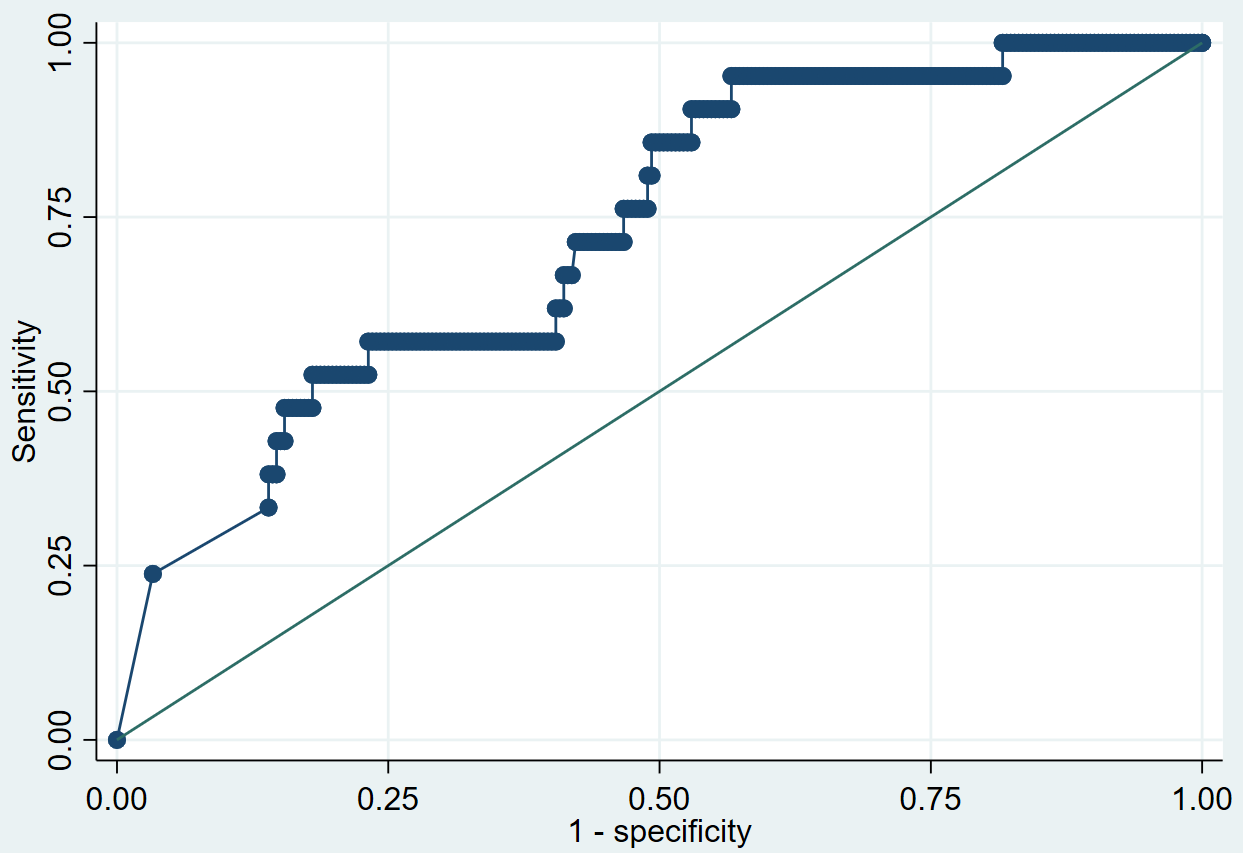


Lactate

AUC = 0.73


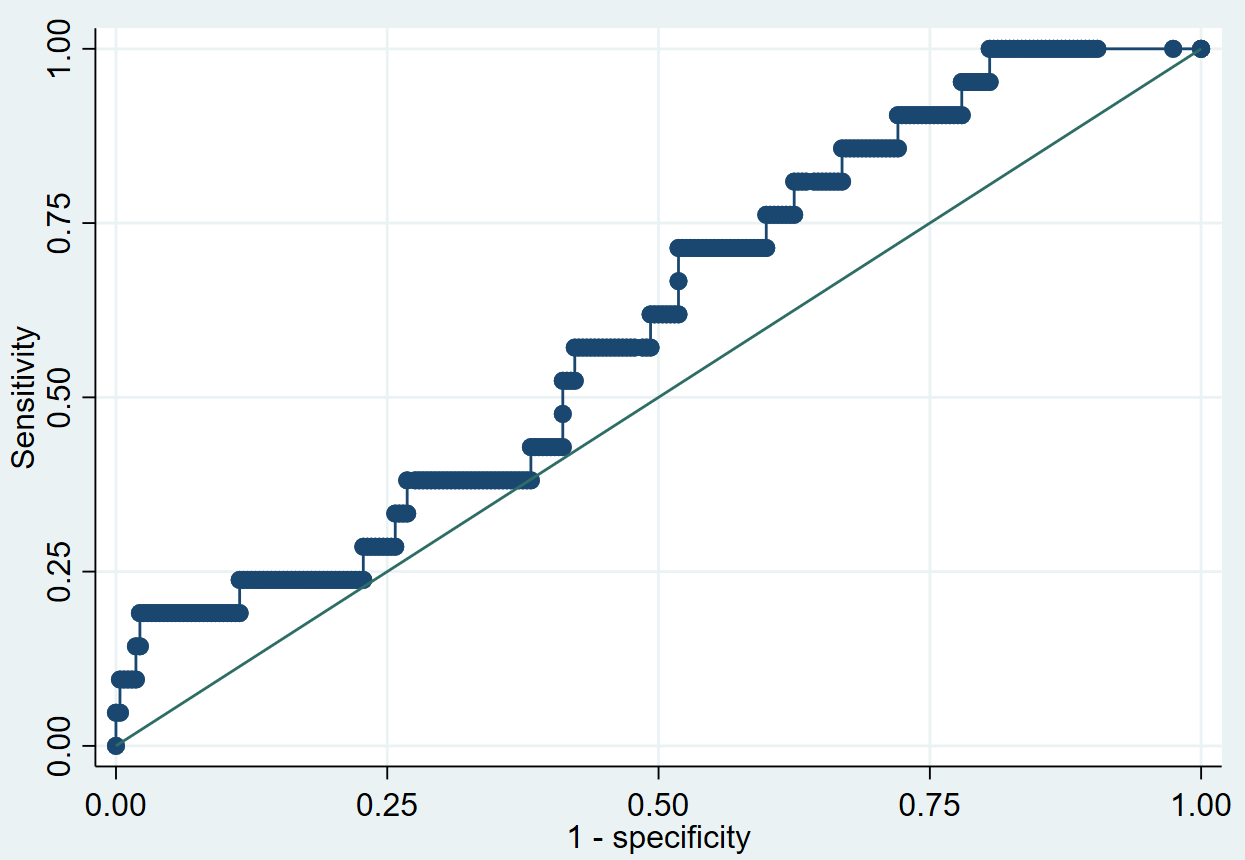


MDA

AUC = 0.61

Notes: Linear discriminant analysis (LDA); Abbreviations: AUC Area Under the Curve, MDA Malondialdehyde
